# Attrition in a telehealth caregiver‐mediated behavioral intervention for children with tuberous sclerosis complex: A mixed‐methods study

**DOI:** 10.1111/dmcn.70384

**Published:** 2026-07-30

**Authors:** Carly Hyde Tillis, Connie Kasari, Nicole McDonald, Monique Vobecky, Maria Pizzano, Jonathan Panganiban, Brandon Gharapetian, Elise Y. Tran, Charles A. Nelson, Elizabeth A. Thiele, Shafali Jeste

**Affiliations:** ^1^ Department of Health Policy and Management University of California Los Angeles California USA; ^2^ Department of Education University of California Los Angeles California USA; ^3^ Semel Institute for Neuroscience University of California Los Angeles California USA; ^4^ Department of Pediatrics and Neurology Children’s Hospital Los Angeles Los Angeles California USA; ^5^ Graduate School of Psychology California Lutheran University Thousand Oaks California USA; ^6^ Department of Pediatrics Boston Children's Hospital and Harvard Medical School Boston Massachusetts USA; ^7^ Department of Neurology Massachusetts General Hospital Boston Massachusetts USA; ^8^ Department of Pediatrics University of California Los Angeles California USA

## Abstract

**Aim:**

To examine factors contributing to attrition in a telehealth, caregiver‐mediated behavioral intervention for young children with tuberous sclerosis complex (TSC).

**Method:**

This randomized waitlist‐control trial (*n* = 59) included children (12–39 months) with TSC and their caregivers. A convergent parallel mixed‐methods design investigated factors influencing attrition. Quantitative analyses used negative binomial regression to explore predictors of session completion; qualitative data were thematically analyzed and integrated with quantitative patterns to complement and expand findings.

**Results:**

Negative binomial regression did not yield a significant overall model and explained limited variance. Two candidate predictors showed exploratory associations with fewer completed sessions: higher seizure severity (incidence rate ratio [IRR] = 0.95, *p* = 0.046) and COVID‐era enrollment (IRR = 0.77, *p* = 0.046). Distance to site and socioeconomic risk were not associated with attrition. Qualitative data reinforced medical crises as primary reasons for attrition and expanded on perceived benefit and other factors contributing to overall caregiver burden.

**Interpretation:**

In this clinical trial, socioeconomic, ethnic, and geographic factors were not associated with attrition, although the study was underpowered to detect small or moderate effects. Families of children facing complex developmental disability may nonetheless require additional adaptation and support to sustain engagement in telehealth caregiver‐mediated interventions. Mixed‐methods integration highlighted the multifaceted nature of attrition and the need for more precise measures to understand it.

## Graphical abstract

Exploratory quantitative analyses suggested that higher seizure severity and COVID‐era enrollment were associated with lower intervention completion, while qualitative findings identified cumulative caregiver burden, medical instability, competing demands, and perceived intervention fit as contributors to attrition. Flexible, adaptive intervention models may help support sustained engagement among families of children with complex developmental disabilities.

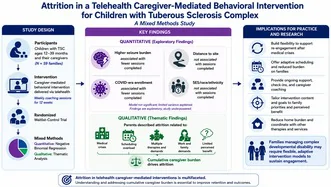

**This original article is commented by Holland on pages** 1499–1500 **of this issue.**

**Abbreviations**
IRR: incidence rate ratio
TSC: tuberous sclerosis complex
JASPER: Joint Attention, Symbolic Play, and Engagement Regulation

**What this paper adds**

- Exploratory modeling suggests that higher seizure burden and COVID‐era enrollment may relate to intervention attrition.
- Distance and socioeconomic/minoritized status were not associated with attrition in this exploratory sample.
- Qualitative findings reinforce competing demands and perceived fit/benefit as additional reasons for withdrawal.
- Mixed‐methods insights underscore the need for careful qualitative and quantitative measurement of attrition factors in clinical trials.

Attrition in intervention studies is more than a logistical or statistical concern; it offers insight into real‐world feasibility and participant engagement. Understanding dropout patterns is essential for optimizing strategies for at‐risk populations and improving effectiveness, outcomes, and equity.^1^, ^2^ Identifying how different populations respond can also guide more personalized and accessible program design.

In pediatric research, attrition is a persistent challenge, commonly associated with minority status, lower socioeconomic status, younger parent age, and single‐parent households.^3^, ^4^, ^5^, ^6^, ^7^ These factors often operate cumulatively; families facing multiple risks are more likely to drop out and show reduced treatment response.^1^ Retention is particularly difficult in behavioral interventions, which require sustained time, emotional effort, and caregiver involvement. Caregiver‐mediated models introduce additional burden because caregivers must deliver the intervention themselves. Parental employment and stress predict dropout,^8^ and study design features such as site or treatment condition also influence retention.^9^

Telehealth models alter these dynamics. Although distance traditionally predicts dropout,^3^, ^4^, ^6^, ^8^ telehealth may reduce this barrier; however, its effect remains underexamined. In two computer‐based interventions, stress, marital status, and socioeconomic status, rather than distance, predicted attrition.^10^, ^11^

Child developmental characteristics, such as level of disability or autism symptoms, generally do not predict attrition, but child health does.^1^, ^8^ For children with complex developmental disability, where medical, neurological, and developmental factors interact,^12^ developmental characteristics are relatively stable, whereas health issues can create acute disruptions that hinder participation. Both low and high disease severity contribute to dropout: milder symptoms may reduce perceived relevance,^13^, ^14^ while severe illness can impede adherence during acute episodes.^11^, ^14^, ^15^

This study examines attrition in a telehealth, caregiver‐mediated intervention for children aged 12 to 36 months with tuberous sclerosis complex (TSC), a rare genetic disorder caused by *TSC1* or *TSC2* mutations.^16^ TSC is often diagnosed in infancy or prenatally because of cardiac or brain hamartomas.^15^, ^17^, ^18^ Epilepsy typically begins in the first year of life,^17^ with up to 60% of children meeting autism criteria and over 80% show cognitive impairment.^19^, ^20^, ^21^ Children with TSC frequently experience complex developmental disability, generating significant care challenges across medical and therapeutic domains.^12^ Caregivers in this trial reported that community providers often misunderstand TSC's impact on behavioral interventions,^22^ highlighting the need for specialized approaches and the importance of this first behavioral intervention trial in this population.

To understand intervention engagement among families of children with TSC, we used a convergent parallel mixed‐methods design to examine quantitative and qualitative predictors of dropout. Our goal was to identify key drivers of attrition and explore unmeasured barriers affecting telehealth engagement in the context of complex developmental disability. Based on prior literature and trial context, we expected multiple factors, including seizure severity, caregiver employment, socioeconomic or minoritized status, and distance, to contribute to dropout. Seizure burden was particularly salient given TSC's early‐onset epilepsy and frequent hospitalizations, which caregivers described as major disruptions.^17^, ^22^ We also hypothesized that pandemic‐related disruptions would influence attrition because of broader family strain and the demands of a caregiver‐mediated model.

## METHOD

### Clinical trial overview

The parent clinical trial was registered with ClinicalTrials.gov (NCT03422367). This analysis was not separately preregistered. Data supporting this study are openly available in the National Database for Autism Research.

### Participants

Participants were children aged 12 to 39 months with a clinical diagnosis of TSC and their primary caregivers. Of 61 enrolled families, two were excluded at screening for developmental levels below eligibility criteria, yielding a final randomized sample of 59. Additional exclusion criteria included planned epilepsy surgery during the trial. Recruitment occurred through specialty clinics, the TSC Alliance, social media, and medical record queries.

### Enrollment and consent

Recruitment and enrollment occurred at two university hospitals (September 2017–May 2023). The study was reviewed and approved by the Institutional Review Board at the University of California, Los Angeles. Caregivers completed in‐person informed consent with a trained coordinator who reviewed all study procedures.

### Randomization

After baseline assessments confirmed eligibility, participants were randomized to immediate treatment or waitlist control using an online system. Block randomization stratified by child age (< 24 months vs ≥24 months) ensured balance. Because of the behavioral nature of the intervention, caregivers and some staff were not blinded. Treatment began immediately for the intervention group and after the third assessment for the waitlist group.

### Baseline assessments

Baseline visits included developmental, autism, and play assessments, parent questionnaires, and electroencephalogram. Adaptive functioning was assessed using the Vineland Adaptive Behavior Scales, Third Edition, a standardized caregiver‐report measure of communication, daily living, socialization, and motor skills.^23^ Families received summary reports (behavioral observations, standardized scores, clinical interpretation). Optional consultations with study clinicians were offered post‐visit.

### Assessment flexibility and retention strategies

Although baseline assessments were in person, the intervention was delivered primarily via telehealth videoconferencing. For clarity, we refer to the overall design as a telehealth caregiver‐mediated intervention and specify ‘in‐person’ procedures when relevant. COVID‐era follow‐ups were completed via telehealth using Zoom for Healthcare; families could choose telehealth or in‐person formats. Visits were targeted within 4 weeks, with rescheduling up to 8 weeks allowed for illness, seizures, hospitalizations, or other conflicts. Retention strategies included flexible scheduling, reminders, and multiple contact methods (phone, email, text). When families were unresponsive, contact methods and timing were adjusted, and alternate family contacts were used when necessary. Financial support for in‐person visits (flight reimbursement and hotel stays) was provided through study grants and private foundations (BeCureful). Cash or gift cards were provided for all assessment visits.

### Behavioral intervention

The 12‐week caregiver‐mediated intervention JASPER (Joint Attention, Symbolic Play, and Engagement Regulation) targeted child social communication, play, and engagement.^24^, ^25^ A designated caregiver participated with coaching from a trained interventionist, and additional caregivers could join. Families were taught naturalistic behavioral strategies. Adapted before COVID‐19 for telehealth access,^26^ it consisted of weekly 45‐minute synchronous telehealth sessions and asynchronous review of caregiver‐submitted practice videos; 67% of weekly sessions were completed via telehealth and the remainder were conducted in person. All but one participant completed at least one in‐person session. The intervention closed at 24 weeks. Participants who had difficulty scheduling or were missing sessions but had not withdrawn were moved to post‐intervention assessments at that time. All randomized participants were included in this attrition analysis.

### Patient and public involvement

Caregivers of children with TSC, as individuals with lived experience, significantly informed study design and implementation, including telehealth intervention adaptation, assessment procedures, and interpretation of attrition findings.

### Quantitative methods

#### Measure selection

Candidate predictors were selected based on prior attrition research, study context, and the Social‐Ecological Model of Engagement.^27^ To accommodate the available sample size, variables considered during initial planning (including demographic and clinical characteristics) were condensed or excluded because of conceptual overlap. Two additional variables with greater than 10% missingness (marital status and caregiver involvement ratings) were removed to preserve complete cases. One participant missing five of the nine analytic variables was excluded. A second participant, missing only household income, had this value imputed using the ZIP‐code median income. The final analytic sample included 58 participants with complete data for all nine predictors.

#### Indicator variables

COVID‐era enrollment (before vs during/after March 2020), study site (A/B), caregiver employment (> 30 hours/week), and group assignment (immediate vs waitlist) were included.

#### Parenting daily hassles

The 20‐item Parenting Daily Hassles Frequency Scale measured frequency of common caregiving stressors; higher scores reflect greater stress.^28^

#### Outside intervention hours

Weekly therapy hours across modalities (speech, physical, behavioral, sensory, developmental) were parent‐reported and averaged annually.

#### Seizure severity score

The seizure severity score was adapted from the Early Childhood Epilepsy Severity Scale using baseline data.^29^ Severity was the sum of: (1) seizure frequency on a 0 to 4 scale (0 = none; 1 = less than weekly/monthly/rare; 2 = weekly; 3 = daily; 4 = more than daily, assessed over 2 months pre‐assessment); (2) number of seizure types; and (3) number of current antiseizure treatments (medications, diets, supplements). Higher scores indicate greater severity.

#### Socioeconomic and minority status

A cumulative score for socioeconomic and minority status was developed for this study. Cumulative risk approaches have previously been used to capture the multidimensional nature of socioeconomic disadvantage and related exposures.^1^, ^30^ Maternal education, household income, and minoritized status were coded to reflect increasing exposure to sociodemographic disadvantage: maternal education (high school/less = 2; associate/trade = 1; college/greater = 0), income (< US$50 000 = 2; US$50 000–90 000 = 1; > US$90 000 = 0), and minority status (non‐White = 1; non‐Hispanic White = 0). Points were summed, with higher scores indicating greater cumulative sociodemographic disadvantage. This study‐specific cumulative approach was used to reduce model complexity while capturing multidimensional sociodemographic disadvantage.

#### Outcome

Session completion was defined as the number of fully attended intervention sessions (0–12), verified through interventionist records. A session was coded as completed if caregiver and interventionist met for 15 minutes or longer. Sessions canceled in advance or missed entirely were coded as not completed.

#### Statistical analysis

Because the outcome was a count variable with significant overdispersion (likelihood‐ratio test of α = 0: χ^2^ = 6.84, *p* = 0.004), negative binomial regression was used. Although session completion was bounded by the study protocol, the distribution remained skewed and overdispersed, making negative binomial regression a pragmatic exploratory modeling approach for this sample. All predictors were entered simultaneously. Results are reported as incidence rate ratios (IRRs) with standard errors, 95% confidence intervals, and model diagnostics (log‐likelihood, pseudo‐*R* ^2^, dispersion parameter). Statistical significance was set at a *p*‐value lower than 0.05. The parent trial was powered to detect treatment effects rather than predictors of attrition; therefore, these analyses should be considered exploratory and hypothesis‐generating, with limited power to detect small or moderate effects. A post hoc reduced model including conceptually central predictors with preliminary associations was estimated to assess robustness but did not replace the theory‐driven primary model.

### Qualitative methods

Qualitative analysis focused on the 12 families who actively withdrew from the intervention; families who reached the end of the intervention period without completing all sessions but remained enrolled were not included. All withdrawal‐related qualitative materials were compiled into a secure database, including coordinator notes, protocol deviation logs, summaries of withdrawal‐related phone calls, emails, and texts. All 12 cases included coordinator‐reported documentation, and five cases also included direct caregiver communications.

An interpretivist, inductive thematic approach was used to identify factors contributing to withdrawal, with the aim of elaborating and contextualizing quantitative findings rather than generating a standalone explanatory model. A five‐code framework for withdrawal reasons was developed, and two trained coders independently applied the codes to all materials. Discrepancies were resolved through discussion, and interrater reliability was excellent (Cohen's κ > 0.94). These codes were then used to develop higher‐level themes. Because withdrawal communications varied in depth and format, data adequacy and saturation were not expected; findings are interpreted as illustrative. Reflexivity was supported through peer debriefing with senior team members familiar with the intervention and clinical context.

### Integration

Integration used a triangulation approach^31^ in which quantitative results were prioritized. Themes from the qualitative analysis were compared with quantitative results to identify areas of convergence, contextualization, and expansion, recognizing that qualitative findings could extend beyond quantitatively supported associations; this integration is presented in the ‘Discussion’.

## RESULTS

### Participant characteristics

Child and caregiver characteristics are described in Table 1. Nearly all participants (94.9%) had epilepsy, with 37.9% currently experiencing seizures (Table 1). Overall, 31.0% were non‐White. Participants resided in 26 US states.

**TABLE 1:** Participant demographics and clinical characteristics for included sample (*n* = 58).

Demographic	*n*	%	Mean	SD
**Child clinical characteristics**				
TSC mutation type				
*TSC1* mutation	2	3.4		
*TSC2* mutation	39	67.8		
Unknown mutation	17	28.8		
Current seizure frequency				
No seizures	36	62.1		
Monthly seizures	8	13.8		
Weekly seizures	3	5.2		
Daily seizures	6	10.3		
More than daily seizures	5	8.6		
Number of seizure types			1.7	1.0
History of infantile spasms	36	62.1		
Number of medications			2.3	1.4
Seizure severity total			4.9	2.9
Outside hours of intervention			3.2	3.8
VABS‐3 composite score			77.3	15.5
**Caregiver characteristics**				
Primary caregiver relationship				
Biological mother	47	81.4		
Adoptive mother	3	5.1		
Father	6	10.2		
Grandmother	2	3.4		
Parenting daily hassles (frequency)			36.1	14.3
SES‐M total score			1.3	1.5
Minoritized status				
Non‐Hispanic White	40	69.0		
Non‐White	18	31.0		
Income				
Income < US$50 000	10	17.2		
Income US$50 000–US$90 000	11	19.6		
Income > US$90 000	37	66.1		
Education				
High school or lower	9	15.5		
Associate/trade school	8	13.8		
College or greater	41	73.2		
COVID‐19	29	50.0		
Employed	32	55.2		
Waitlist status	29	50.0		
Site A	34	58.6		
Miles from study site			531.0	586.3
Number of intervention sessions			8.9	4.0

Abbreviations: SES‐M, socioeconomic and minority status; TSC, tuberous sclerosis complex; VABS, Vineland Adaptive Behavior Scales, Third Edition.

### Intervention completion

Of 59 randomized participants, 57 initiated the intervention (Figure S1). Families completed an average of 8.9 sessions (SD = 5.3), with higher completion among immediate‐treatment participants than waitlist participants. Session‐completion distribution and ratings of caregiver involvement are presented in Appendix S1. Adverse events unrelated to the study or intervention were reported in 17 cases of illness, 16 hospitalizations (including two for unplanned epilepsy surgery), and 11 instances of seizure worsening.

### Quantitative results

Negative binomial regression was used to examine predictors of session completion. The overall model was not statistically significant (LR χ^2^[9] = 12.96, *p* = 0.165) and explained a small proportion of variance (pseudo‐*R* ^2^ = 0.0369). This indicates that the full predictor set did not explain more variance in session completion than an intercept‐only model. For this reason, the findings should be interpreted as exploratory and hypothesis‐generating patterns rather than definitive effects.

As shown in Figure 1, two candidate predictors showed small exploratory associations with completing fewer sessions: higher baseline seizure severity (IRR = 0.95, *p* = 0.046) and COVID‐era enrollment (IRR = 0.77, *p* = 0.046). Other predictors, including prior therapy hours, caregiver employment, socioeconomic and minority status, distance to site, and study location, were not associated with session completion.

**FIGURE 1:**
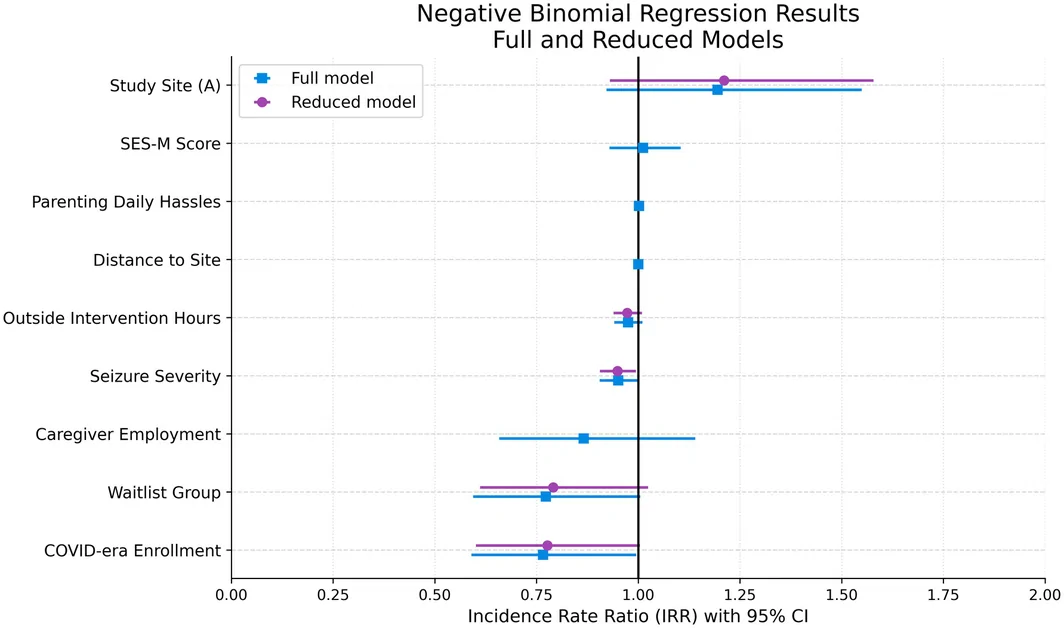
Incidence rate ratios (IRR) and 95% confidence intervals (CIs) from the full and reduced negative binomial models predicting the number of sessions completed. Abbreviation: SES‐M, socioeconomic and minority status.

The reduced model showed a similar pattern of associations, although it still did not reach significance (LR χ^2^[5] = 10.89, *p* = 0.054). The consistency of coefficients across both models suggests that the observed patterns were stable within the constraints of the study's small sample size (Figure 1). Full IRR tables and 95% confidence intervals for models are provided in Appendix S2.

### Qualitative results

Thematic analysis identified five themes describing attrition factors and process.

Competing demands, scheduling conflicts, and life events were most common. Many families reported difficulty balancing the intervention with work, therapies, and other daily demands. As one parent shared, ‘[CHILD] has a lot of services on him right now and it will be too much adding this on’. Overloaded schedules, especially during COVID‐era enrollment, often led to cancellations and inconsistent participation. Acute disruptions like moving, international travel, or housing instability created logistical barriers.

Medical conflicts also significantly contributed to attrition. Children experienced hospitalizations or acute medical events, often leading to extended communication gaps. Caregivers reported feeling overwhelmed or unable to resume the study during/after these crises.

Perceived lack of fit or benefit was cited by caregivers who felt the intervention did not meet their family's needs or yield enough observable benefit. As one participant expressed, ‘I don't feel like participating in the study is working for our family’.

Conditional disengagement emerged in cases where caregivers indicated openness to rejoining. Two families withdrew because of adequate service provision elsewhere but expressed interest in resuming participation if circumstances changed. These cases suggest dropout may reflect timing or perceived need, rather than permanent disengagement.

Cumulative burden was frequently reported by families experiencing stressors in combination. As one caregiver noted, ‘Mine and my son's schedule has become extremely busy with doctor appointments, therapies, etc. I have not seen the benefit of [the intervention] yet and unfortunately feel we should probably pull out of the study at this time’, illustrating compounding demands and multiple reasons for withdrawal.

## DISCUSSION

This mixed‐methods study examined predictors of attrition in a telehealth behavioral intervention for young children with TSC. Although quantitative models were underpowered, qualitative findings clarified mechanisms of dropout and highlighted factors not captured quantitatively, offering guidance for future TSC trials and telehealth delivery for families managing complex developmental disability.

### Convergence of findings

Greater seizure severity was associated with lower session completion in exploratory analyses, while qualitative reports similarly described hospitalizations and health crises as major barriers to participation. This non‐random attrition is concerning, as it suggests systematic underrepresentation of children with more severe epilepsy^32^ and limited generalizability, with implications for trials involving young children with complex developmental disability.

### Expansion of quantitative findings

While underpowered, socioeconomic and minority status and distance were not associated with attrition in the current sample. Although these findings may indicate that telehealth delivery reduces some traditional access barriers, they should be interpreted cautiously given the study's limited power to detect small or moderate effects. Nonetheless, the study enrolled families representing a range of sociodemographic backgrounds and geographic regions,^26^ suggesting that telehealth delivery may support broader participation in intervention research for families of children with TSC.

Qualitative data showed that some families disengaged when they were already receiving adequate services elsewhere. This may reflect a range of factors, including perceived intervention fit, positive experiences with other programs, provider recommendations, or preferences for in‐person versus telehealth delivery when multiple options were available. Caregivers also cited limited perceived benefit as a reason for attrition, a central factor in adherence literature that is not measured here.^2^, ^33^ Together, these findings highlight the importance of further examining how families evaluate telehealth interventions relative to other available services, including considerations related to home implementation demands, flexibility, rapport, and perceived benefit. Although telehealth delivery may reduce geographic barriers, reducing travel burden alone may not eliminate broader caregiving and participation demands for families managing complex developmental disability. It will be important to ensure that telehealth interventions remain flexible, feasible, and responsive to family needs in order to avoid unintentionally reinforcing disparities in participation or engagement. Incorporating early expectation and satisfaction ratings may improve also assessment of intervention fit.

Parental employment, parental stress, and outside therapy hours were not significant quantitative predictors, yet qualitative data emphasized scheduling conflicts, competing demands, and life events as common drivers of withdrawal. Qualitative findings suggested that attrition often reflected intersecting and compounding stressors rather than any single isolated factor, with families frequently describing combinations of medical instability, competing therapies, employment demands, scheduling difficulties, and perceived intervention fit contributing simultaneously to withdrawal decisions. These patterns are consistent with a cumulative burden framework,^1^, ^34^ in which dropout reflects intersecting stressors rather than single predictors. The quantitative finding that COVID‐era enrollment was associated with lower session completion may similarly reflect heightened caregiving and work demands during the pandemic, though the study was not designed or powered to directly test a multidimensional cumulative burden model quantitatively.^35^ Future research may benefit from prospective measurement approaches, such as composite caregiver‐burden indices, that better capture intersecting and evolving caregiver and clinical pressures over time.

### Limitations

This study's quantitative model was not significant overall, reflecting both limited statistical power (small *n* relative to the number of predictors) and likely influence of unmeasured factors. Quantitative findings should therefore be interpreted as exploratory and hypothesis‐generating rather than confirmatory. Additionally, the COVID‐era indicator combined multiple phases of the pandemic and recovery period, including periods with differing public health restrictions and healthcare disruptions. The sample size did not support finer‐grained temporal analyses, and future studies may benefit from examining these periods separately. Qualitative data were limited by small sample size, lack of saturation, and potential response bias from families who completely disengaged. Structured exit interviews could add contextual depth but would remain vulnerable to missing data from families who disengaged entirely. Additionally, qualitative convergence should not be interpreted as confirmation of statistically weak quantitative associations but rather as contextualization and expansion of patterns observed in exploratory analyses. Overall, generalizability is constrained by the telehealth caregiver‐mediated design and the population with TSC, though findings may extend to other groups with complex developmental disability. Lack of blinding in caregiver‐mediated trials may also introduce observer or engagement bias. Future trials may benefit from incorporating a cumulative caregiver burden index administered longitudinally throughout participation, reducing the number of individual predictors while better capturing dynamic, intersecting stressors.

### Implications for engagement

Families of children with complex developmental disability may require flexible clinical trial and intervention designs that anticipate disruptions, support re‐engagement, and account for fluctuating caregiver and medical burden. Practical considerations emerging from the current study are summarized in Table 2, including strategies related to medical instability, competing caregiver demands, perceived intervention fit, communication during periods of disengagement, and telehealth‐related home implementation burden. Without such adaptations, research and intervention models risk excluding families facing the greatest caregiving and health‐related challenges.

**TABLE 2:** Practical considerations for telehealth behavioral intervention trials involving children with complex developmental disability.

Domain	Challenges observed	Potential strategies
Medical instability	Hospitalizations, seizure worsening, and acute health events disrupted participation	Allow pauses and flexible re‐entry; offer lower‐intensity participation during crisis
Competing caregiver demands	Therapies, work, appointments, and caregiving demands contributed to overload	Flexible scheduling; simplified and adaptable home practice; ‘maintenance weeks’ during high‐burden periods
Perceived fit and benefit	Some families no longer felt the intervention was useful or feasible	Use collaborative goal‐ and expectation‐setting; revisit perceived benefit and fit; tailor coaching to family priorities
Communication and re‐engagement	Families sometimes disengaged temporarily during periods of stress	Use supportive check‐ins, normalize interruptions, and create low‐pressure re‐engagement pathways
Telehealth access and home‐based burden	Telehealth reduced travel demands but did not eliminate scheduling, caregiving, and home‐practice burden	Assess caregiver capacity throughout participation; reduce non‐essential home‐practice burden; offer flexible formats when feasible

### Conclusion

Findings suggest that telehealth may support broader access to intervention research while also underscoring the influence of health instability and cumulative caregiver burden on attrition. Mixed‐methods approaches remain essential for understanding dropout and refining measurement to support inclusive, adaptive intervention and trial designs for children with complex developmental disability.

## CONFLICT OF INTEREST STATEMENT

The authors declare no conflicts of interest.

## Supporting information


**Figure S1:** CONSORT diagram for participant flow in the clinical trial

**Appendix S1:** Intervention completion details

**Appendix S2:** Negative binomial regression model results

## Data Availability

The data that support the findings of this study are openly available in the National Database for Autism Research (NDAR) at https://catalog.data.gov/dataset/national‐database‐for‐autism‐research‐ndar.
