# Supplementary material for: Attrition in a telehealth caregiver‐mediated behavioral intervention for children with tuberous sclerosis complex: A mixed‐methods study
Source: Dev Med Child Neurol. 2026 Jul 30;68(11):1621–9. doi: 10.1111/dmcn.70384 (PMC13649290; doi:10.1111/dmcn.70384)
Supplement: Supplementary file 1 [file DMCN-68-1621-s001.pdf]

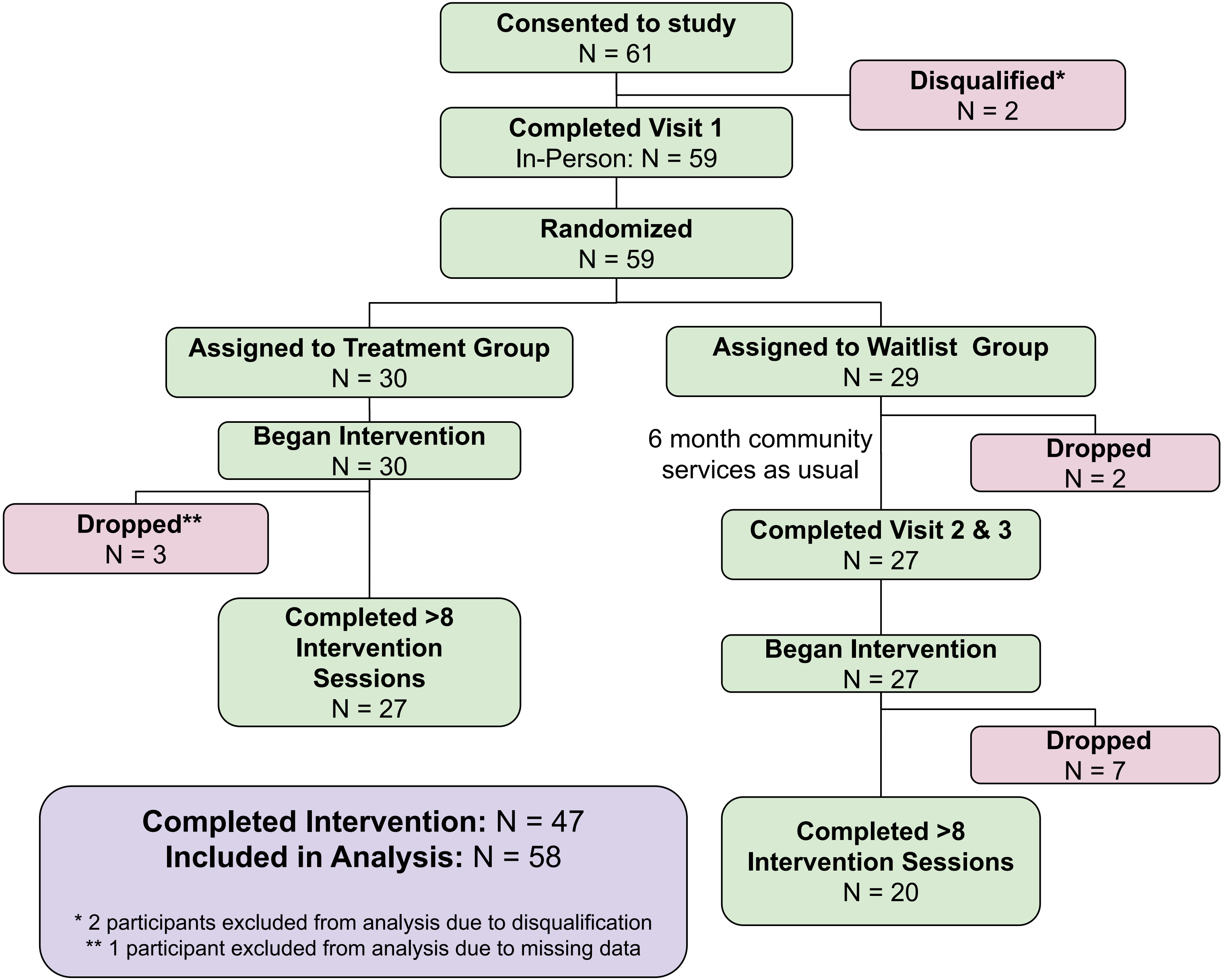

Consented to study
N = 61
Disqualified*
N = 2
Completed Visit 1
In-Person: N = 59
Randomized
N = 59
Assigned to Treatment Group
N = 30
Assigned to Waitlist Group
N = 29
Began Intervention
N = 30
6 month community services as usual
Dropped
N = 2
Dropped**
N = 3
Completed Visit 2 & 3
N = 27
Completed >8 Intervention Sessions
N = 27
Began Intervention
N = 27
Dropped
N = 7
Completed Intervention: N = 47
Included in Analysis: N = 58
* 2 participants excluded from analysis due to disqualification
** 1 participant excluded from analysis due to missing data
Completed >8 Intervention Sessions
N = 20
