# Supplementary material for: Attrition in a telehealth caregiver‐mediated behavioral intervention for children with tuberous sclerosis complex: A mixed‐methods study
Source: Dev Med Child Neurol. 2026 Jul 30;68(11):1621–9. doi: 10.1111/dmcn.70384 (PMC13649290; doi:10.1111/dmcn.70384)
Supplement: Supplementary file 2 [file DMCN-68-1621-s002.docx]

**Appendix S2: Negative Binomial Regression Model Results**

*Overall Model Fit.* The primary Negative Binomial Regression model examining predictors of session completion was not statistically significant overall (Likelihood Ratio χ²(9) = 12.96, p = .165), with a pseudo R² of .0369. The likelihood-ratio test of alpha indicated significant overdispersion (χ² = 6.84, p = .004), supporting the use of Negative Binomial rather than Poisson regression.

A post-hoc exploratory reduced model including fewer predictors showed slightly improved overall fit but remained nonsignificant (Likelihood Ratio χ²(5) = 10.89, p = .054), with a pseudo R² of .0310. The alpha test again supported NBreg over Poisson (χ² = 8.45, p = .002).

**Table S1. Full Model: Incidence Rate Ratios (IRR) for Predictors of Session Completion (N = 58)**

| **Predictor** | **IRR** | **SE** | **p-value** | **95% CI** |
| --- | --- | --- | --- | --- |
| Post-COVID enrollment | 0.766 | 0.102 | 0.046 | 0.590 – 0.995 |
| Seizure Severity | 0.951 | 0.024 | 0.046 | 0.905 – 0.999 |
| Waitlist Status | 0.773 | 0.103 | 0.054 | 0.594 – 1.005 |
| Prior Therapy Hours | 0.975 | 0.018 | 0.162 | 0.941 – 1.010 |
| Caregiver Employment | 0.866 | 0.121 | 0.304 | 0.658 – 1.140 |
| SES-R Score | 1.012 | 0.045 | 0.781 | 0.929 – 1.104 |
| Distance to Site (Miles) | 1.000 | 0.0001 | 0.540 | 0.9998 – 1.0003 |
| Study Site (UCLA) | 1.195 | 0.158 | 0.179 | 0.922 – 1.549 |
| Parent Distress History | 1.002 | 0.006 | 0.664 | 0.992 – 1.013 |

*Full Model statistics.*
LR χ²(9) = 12.96, p = .165
Pseudo R² = .0369
Alpha = 0.108 (p = .004)

**Table S2. Reduced Model: Incidence Rate Ratios (IRR) for Predictors of Session Completion (N = 58)**

| **Predictor** | **IRR** | **SE** | **p-value** | **95% CI** |
| --- | --- | --- | --- | --- |
| Study Site (UCLA) | 1.211 | 0.163 | 0.156 | 0.930 – 1.578 |
| Prior Therapy Hours | 0.973 | 0.018 | 0.145 | 0.939 – 1.009 |
| Seizure Severity | 0.949 | 0.022 | 0.027 | 0.906 – 0.994 |
| Waitlist Status | 0.791 | 0.104 | 0.075 | 0.611 – 1.024 |
| Post-COVID Enrollment | 0.777 | 0.102 | 0.054 | 0.601 – 1.004 |

*Model statistics.*
LR χ²(5) = 10.89, p = .054
Pseudo R² = .0310
Alpha = 0.122 (p = .002)
