# Supplementary material for: Attrition in a telehealth caregiver‐mediated behavioral intervention for children with tuberous sclerosis complex: A mixed‐methods study
Source: Dev Med Child Neurol. 2026 Jul 30;68(11):1621–9. doi: 10.1111/dmcn.70384 (PMC13649290; doi:10.1111/dmcn.70384)
Supplement: Supplementary file 3 [file DMCN-68-1621-s003.docx]

**Appendix S1: Intervention Completion Details**

*Participant Flow and Intervention Initiation.* Of the 59 randomized participants, 57 initiated the intervention. This included 30 participants in the immediate treatment group and 27 in the waitlist group.

*Intervention Completion Rates.* Across the analytic sample, the mean number of completed sessions was 8.90 (SD = 4.01; range: 0–12). Children in the immediate-treatment group completed a mean of 9.86 sessions (SD = 2.90), and those in the waitlist group completed a mean of 7.93 sessions (SD = 4.75). The distribution of completed sessions is presented in Table A1.

*Caregiver Quality of Intervention (CQI) Score.* The CQI is a 4-item observational rating of caregiver implementation quality completed by clinicians after each session. Items on caregiver comfort, confidence, enthusiasm/interest, accuracy are rated on a 1–5 scale, with higher scores reflecting stronger caregiver use of intervention strategies. CQI scores were calculated only for participants with at least two sessions attended (N = 50), allowing an average score to be computed. Among these participants, the mean CQI composite score was 3.5 (SD = 0.4), with observed values ranging from 2.4 to 4.6.

**Distribution of Completed Intervention Sessions (N = 59)**
